# Severe anaemia associated with *Plasmodium falciparum* infection in children: consequences for additional blood sampling for research

**DOI:** 10.1186/s12936-016-1356-9

**Published:** 2016-06-02

**Authors:** Laura Maria Francisca Kuijpers, Jessica Maltha, Issa Guiraud, Bérenger Kaboré, Palpouguini Lompo, Hugo Devlieger, Chris Van Geet, Halidou Tinto, Jan Jacobs

**Affiliations:** Department of Clinical Sciences, Institute of Tropical Medicine, Antwerp, Belgium; Department of Microbiology and Immunology, KU Leuven, Leuven, Belgium; Centre for Molecular and Vascular Biology, Leuven, Belgium; Institut de Recherche En Sciences de La Santé (IRSS/Clinical Research Unit of Nanoro (CRUN), Nanoro, Burkina Faso; Paediatrics, University Hospital Leuven, Leuven, Belgium

**Keywords:** Child, Blood specimen collection, Blood volume, Practice guidelines as topic

## Abstract

**Background:**

*Plasmodium falciparum* infection may cause severe anaemia, particularly in children. When planning a diagnostic study on children suspected of severe malaria in sub-Saharan Africa, it was questioned how much blood could be safely sampled; intended blood volumes (blood cultures and EDTA blood) were 6 mL (children aged <6 years) and 10 mL (6–12 years). A previous review [Bull World Health Organ. 89: 46–53. 2011] recommended not to exceed 3.8 % of total blood volume (TBV). In a simulation exercise using data of children previously enrolled in a study about severe malaria and bacteraemia in Burkina Faso, the impact of this 3.8 % safety guideline was evaluated.

**Methods:**

For a total of 666 children aged >2 months to <12 years, data of age, weight and haemoglobin value (Hb) were available. For each child, the estimated TBV (TBVe) (mL) was calculated by multiplying the body weight (kg) by the factor 80 (ml/kg). Next, TBVe was corrected for the degree of anaemia to obtain the functional TBV (TBVf). The correction factor consisted of the rate ‘Hb of the child divided by the reference Hb’; both the lowest (‘best case’) and highest (‘worst case’) reference Hb values were used. Next, the exact volume that a 3.8 % proportion of this TBVf would present was calculated and this volume was compared to the blood volumes that were intended to be sampled.

**Results:**

When applied to the Burkina Faso cohort, the simulation exercise pointed out that in 5.3 % (best case) and 11.4 % (worst case) of children the blood volume intended to be sampled would exceed the volume as defined by the 3.8 % safety guideline. Highest proportions would be in the age groups 2–6 months (19.0 %; worst scenario) and 6 months–2 years (15.7 %; worst case scenario). A positive rapid diagnostic test for *P. falciparum* was associated with an increased risk of violating the safety guideline in the worst case scenario (p = 0.016).

**Conclusions:**

Blood sampling in children for research in *P. falciparum* endemic settings may easily violate the proposed safety guideline when applied to TBVf. Ethical committees and researchers should be wary of this and take appropriate precautions.

## Background

Hospitalized children in sub-Saharan Africa are frequently anaemic due to multiple causes, amongst which severe malaria caused by *Plasmodium falciparum* infection stands out [2, 3]. Concerns may arise when clinical or diagnostic studies are considered in this patient group, in particular when they involve sampling of additional blood. Indeed, as the oxygen binding capacity of blood would further decrease upon sampling of additional blood, the question of how much volume of blood can be sampled safely arises.

Several guidelines for safe blood withdrawal in children exist but they are mainly from institutions based in the USA and there is no general consensus. In a recent review on this subject, Howie concluded that beyond the neonatal period, sampling 3.8 % of total blood volume (equal to 3 ml/kg) over 24 h would be safe [1]. However, he also mentioned that caution should be taken in children with illnesses that impair the replenishment of blood volume or haemoglobin (Hb) [1]. It remains unclear how researchers should interpret this caution when planning studies that involve additional blood sampling in anaemic children.

When planning a diagnostic study on children suspected of severe malaria in sub-Saharan Africa, the authors decided, as part of the study preparation, to assess this 3.8 % safety guideline for its consequences through a desk-based simulation study. From a retrospective cohort of children hospitalized for fever in Burkina Faso, data about weight and Hb value were available; a large proportion of them were anaemic [4]. The estimated total blood volume of the children was corrected for the degree of anaemia. Next, the volumes of blood that were intended to be sampled as part of the planned study were matched to the 3.8 % safety guideline and the proportion of violations (exceeding) of the safety limit were calculated.

## Methods

### The planned study

The planned diagnostic study addressed the differential diagnosis of severe malaria *versus* bacteraemia in children in the Democratic Republic of the Congo. The total volume to be sampled was 6 mL in children <6 years (4 mL for blood culture and 2 mL EDTA blood) and 10 mL for children ≥6 years old (4 mL for blood culture and 6 mL EDTA blood). Based on the estimated total blood volume (TBVe) calculated with the weight for age table of the World Health Organization [5], these volumes were initially considered to be within the 3.8 % safety guideline. The small volume (<0.05 mL) of capillary blood sampled for malaria diagnosis was not taken into account.

### The Burkina Faso study cohort used to assess the safety guideline

The study cohort consisted of all children admitted to a rural hospital in Nanoro, Burkina Faso in whom the frequency of severe malaria and bacteraemia was studied [4]. Inclusion criteria of the original study were an age of <15 years old and presentation with an axillary temperature >38.0 ℃ or with clinical signs of severe illness. Exclusion criteria were unwillingness or inability to give informed consent and neurological symptoms due to a traumatic cause.

From July 2012 to July 2013, data of 711 patients had been collected, including age, weight and Hb concentration (g/dL), determined with a Sysmex XS1000i cell counter (System Corporation, Kobe, Japan). For the purpose of this analysis, two children for whom weight was not recorded were excluded as well as children <1 month of age (n = 15), as the recommended safety guideline is defined for children in the ‘post-neonatal period’. In line with the reference age groups for Hb reference values [6], which set age of 12 years as an upper boundary, children above this age (n = 20) were excluded. In addition, children aged 1–2 months (n = 8) were excluded as they have a more variable and generally higher total blood volume (TBV) (105 mL/kg) compared to older children [1, 7]. The final cohort consisted of 666 children aged >2 months–12 years, admitted with fever and clinical signs compatible with severe malaria and/or bacteraemia. Severe malaria was diagnosed in 292 (41.1 %) children, including 8 (2.7 %) who also had an invasive bacterial infection (bacteraemia and meningitis). Invasive bacterial infection was demonstrated in 67 (9.7 %) children [4].

### Definitions of anaemia and *Plasmodium falciparum* malaria

Anaemia and severe anaemia were defined as Hb < 11 and < 5 g/dL, respectively [8]. Current falciparum malaria infection was defined as *P. falciparum* asexual parasitaemia at microscopy [9]. Recent malaria was defined as negative microscopy but a positive *P. falciparum*-specific histidine-rich protein-2 (HRP2) based rapid diagnostic test (RDT), as HRP2 may persist for weeks after cleared *P. falciparum* infection [9]. Severe malaria was defined as microscopically confirmed malaria and fulfillment of at least one of the WHO clinical or laboratory criteria of severe malaria [10] with slight adaptations: respiratory distress was defined as abnormal deep breathing, subcostal retraction or tachypnea according to age [11]. Malaria transmission in Nanoro is seasonal and hyperendemic [4].

### Quality control

Giemsa-stained thick blood films and strains isolated were stored for retrospective monitoring. Pictures of malaria rapid diagnostic tests were taken at the correct reading time and stored. A total of 10 % of thick blood film slides were reassessed by expert reading.

For the Sysmex measurement of Hb, internal controls and calibrations were done according to the manufacturer’s instructions. The Clinical Research Unit of Nanoro (CRUN) participated in the external quality control assessment organized by UK National External Quality Assessment Service (UK NEQAS) for Haematology (3 monthly assessments) and the National Institute of Communicable Diseases (NICD), South African Republic for Parasitology (3 monthly assessments).

### Calculating estimated and functional total blood volume

For each child, the TBVe (mL) was calculated by multiplying the body weight (kg) by 80 (mL/kg), which is the accepted factor beyond the neonatal period [1, 7]. Next, the TBVe was corrected for the degree of ‘dilution’ by anaemia to obtain the functional TBV (TBVf), meaning the TBV corresponding to a normal oxygen carrying capacity per volume. The correction factor consisted of the rate ‘actual Hb of the child divided by the reference Hb value’, represented as:

$${\text{TBVf }} = \;\frac{{{\text{TBVe }} \times {\text{ actual Hb}}}}{\text{reference Hb per age group}}$$

**Fig. 1:**
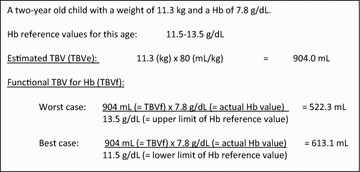
Example of calculating estimated total blood volume (TBVe) and functional total blood volume (TBVf)

Reference Hb values per age group were obtained from the book *First Aid for the Pediatric Clerkship* that was chosen as it mentions reference Hb values with ranges for different age categories in infants [6]. The following age groups were considered: (i) 2–6 months; (ii) 6 months–2 years; (iii) 2–6 years; and (iv) 6–12 years (Table 1). Both the upper and lower limits of the reference Hb values were used to obtain the TBVf for the ‘worst’ and ‘best’ case scenarios, respectively. Figure 1 provides an example of a calculation.

**Table 1:** Reference values of haemoglobin concentration (Hb) per age group used in the present study [6] as well as median reference values obtained from studies in Tanzania [12] and Uganda [12]

Age	Present study		Uganda	Tanzania
	Hb (g/dL) range	Age	Hb (g/dL) median	Hb (g/dL) median
1–2 months	10.0–18.0	<1 year	10.0	10.7
2–6 months	9.5–14.0			
6 months–2 years	10.5–13.5	≥1 to <5 years	10.8	11.3
2–6 years	11.5–13.5	≥5 to <13 years	11.8	12.6
6–12 years	11.5–15.5

### Applying the 3.8 % safety guideline to the blood volumes intended to be drawn

Next, the exact volume that a 3.8 % proportion of this TBVf would represent was calculated for each child (according to the best and the worst case scenario) and this volume was compared to the blood volumes that were intended to be sampled. In children in whom 3.8 % of the TBVf was lower than the blood volumes intended to be drawn, the safety guideline was considered to be violated. If, for instance 3.8 % of the TBVf represented 5 mL for a 14-month old child (for whom 6 mL was intended to be sampled), the 3.8 % safety limit was considered to be violated.

Data were analysed for significance using the Wilcoxon signed-rank test or Chi square test as applicable. A significance level of 0.05 was used.

The original study cohort was obtained as part of a study that was approved by the national ethics committee of Burkina Faso, the institutional review board of the Institute of Tropical Medicine, Antwerp and the ethics committee of the University Hospital of Antwerp [4].

## Results

**Fig. 2:**
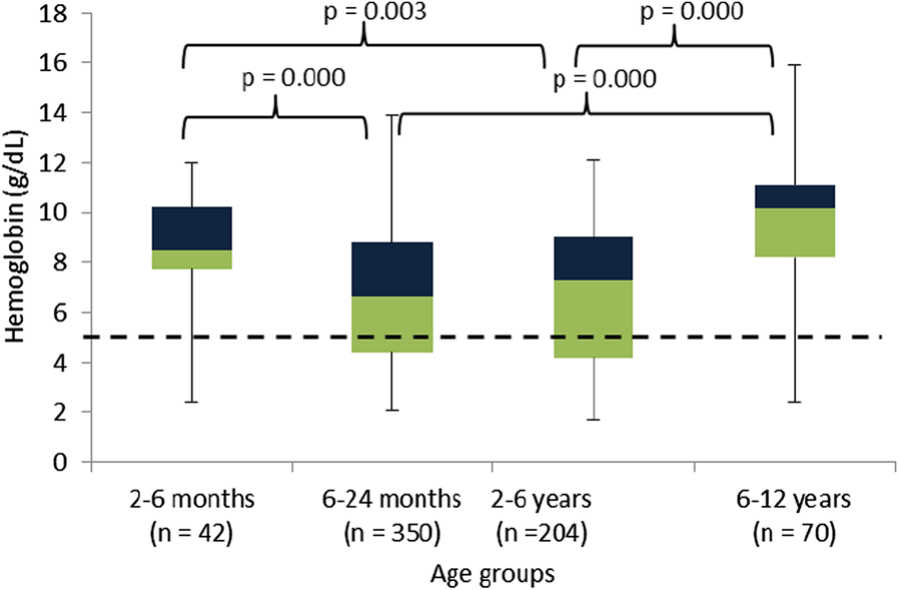
*Box plot* showing the distribution of haemoglobin (Hb) values for the different age groups. The *dotted line* represents the boundary of severe anaemia (Hb value <5 g/dL)

The distribution of the children’s Hb values are displayed per age group in Fig. 2. Table 2 shows the distribution of the TBVe and TBVf for best and worst case scenarios. For all age groups, TBVf was substantially lower than TBVe; differences were largest in the age groups 6–24 months and 2–6 years.

**Table 2:** **The distribution of estimated and functional total blood volumes per age group**

	2–6 months (n = 42)			6–24 months (n = 350)			2–6 years (n = 204)			6–12 years (n = 70)		
	TBVe	TBVf best	TBVf worst	TBVe	TBVf best	TBVf worst	TBVe	TBVf best	TBVf worst	TBVe	TBVf best	TBVf worst
Median	424	393	253	600	364	283	840	480	408	1400	1127	836
Minimum	192	101	68	256	86	67	504	146	124	648	250	185
25th percentile	344	322	185	520	243	189	720	310	264	1200	835	619
75th percentile	468	452	306	680	499	388	960	694	591	1600	1522	1129
Maximum	624	624	411	984	891	693	1760	1262	1075	2240	2242	1664

The age groups and the distribution of estimated and functional total blood volumes (TBVe and TBVf, expressed in mL) for the worst and best case scenario (see text and Fig. 1 for explanation). For convenience of readability, numbers were rounded

**Fig. 3:**
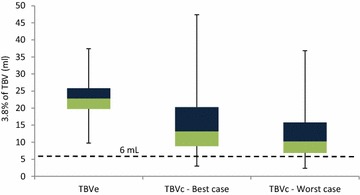
*Box plot* distribution of the actual volume (in mL) that represents the 3.8 % of total blood volume used as safety guideline for children aged 6–24 months (n = 350). Distributions are shown for the estimated and functional total blood volume (TBVe and TBVf), best and worst case scenario. The *dotted line* shows the intended blood volume to be drawn as part of a diagnostic study. Below the *dotted line* are the children for whom the 3.8 % safety guideline would have been violated

Overall, the 3.8 % safety guideline would have been violated in 5.3 % (35/666) and 11.4 % (76/666) of children (best and worst case scenario, respectively). This was most apparent in the age groups 2–6 months and 6–24 months (19 % (8/42) and 15.7 % (55/350) of children for the worst case scenario; see Fig. 3). The other age groups were less affected with, for the worst case scenario, violation of the safety limit in 8.8 % (12/204) and 1.4 % (1/70) of children aged 2–6 years and aged 6–12 years, respectively.

Calculations were also done with the median haemoglobin values found in previous studies in Tanzania [12] and Uganda [13] (Table 1). These median values are similar to the lower limit Hb reference values that were used for the present study. When the calculations were done with the Tanzanian median Hb reference values, the safety guideline would have been violated in 19/666 children (2.9 %). When the calculations were done with the Ugandan median Hb reference values, the safety guideline would have been violated in 32/666 children (4.8 %). Of note, if the safety limit was applied on the TBVe, the intended blood volumes would not have violated the 3.8 % safety guideline in any of the children.

As expected, violation of the 3.8 % safety guideline was related to the high proportion of anaemia in the cohort: 91.4 % (609/666) and 27.6 % (184/666) of children had anaemia and severe anaemia, respectively, with lowest Hb in children aged 6–24 months and 2–6 years (Fig. 2). Severe anaemia in turn was associated with recent or current falciparum malaria, which in turn coincided with the rainy season and shortly thereafter. When the worst-case scenario was considered, the proportion of children in whom the safety guideline would have been violated was 13.2 % (65/494) in the RDT-positive group *versus* 6.4 % (11/172) in the RDT-negative group (p = 0.016). For the best-case scenario, this difference was not statistically different.

Although in the present study severe anaemia was associated with a positive RDT, the RDT result by itself did not accurately predict possible violation of the safety guideline. For 429 out of 494 children (86.9 %) with a positive RDT, the safety guideline would not have been violated.

## Discussion

In this simulation study, it was demonstrated that, in children in a malaria-endemic setting, the safety guideline of 3.8 % of TBV drawn over 24 h would have been frequently violated when considering the TBVf and intended blood volumes of 6 and 10 mL as part of a planned diagnostic study. By contrast, using the estimated TBVe (i.e., not corrected for anaemia), the safety limit would have been violated in none of the children.

Violations were related to high proportions of severe anaemia, which was in turn associated with *P. falciparum* infection. These findings suggest that (at least in malaria-endemic settings) the 3.8 % safety guideline proposed by Howie should be modified, taking into account the TBVf rather than the TBVe. Howie indeed urged for caution when applying this guideline in children with impaired replenishment of blood volume or Hb [1].

Several limitations to the present approach should be considered. First, the 3.8 % safety guideline proposed by Howie was based on existing practices rather than on study evidence [1]. The author further only retrieved two studies that attempted to assess the impact of blood sampling for research in children; both studies, however, included only a few children and those included suffered from rare and specific diseases (children receiving chemotherapy and children with precocious puberty) [14, 15]. Meanwhile, no new studies on this issue has been published since the review by Howie.

Second, the 3.8 % safety guidelines was applied to the TBVf, which is only a proxy for the actual oxygen binding capacity. Moreover, the Hb reference values used might differ from reference values at the study site. Only a few studies have established haematological reference values in children in sub-Saharan Africa [12, 13, 16–18]. They reported differences in reference Hb values compared to industrialized countries and among different geographical sites within sub-Saharan Africa [12–16]. For this reason, calculations were also performed with the median Hb reference values as found in individual studies from Uganda and Tanzania (neighbouring countries of the intended study site). The results obtained with these calculations were similar to the results obtained in what was defined as the best-case scenario.

Intensity and seasonality of falciparum malaria may also be of influence; in the present Burkina Faso cohort, malaria transmission was seasonal and hyperendemic but actual Hb values and proportions of severe anaemia may be different in settings with different patterns and intensity of malaria transmission.

Finally, the clinical impact of the blood volume sampled could not be assessed. The design of the Burkina Faso study included drawing of blood cultures and EDTA-anti-coagulated blood at admission, but clinical and laboratory follow-up, including administration of blood transfusions, were not recorded [4].

Despite these limitations, it was shown that the blood volume safety guideline of 3.8 % may easily be violated in children in *P. falciparum* endemic areas in sub-Saharan Africa. Ethical committees and researchers should be wary of this and take appropriate precautions when considering drawing additional blood for research purposes. First, the volume of blood already sampled as part of standard (routine) patient care needs to be established, particularly since in most resource-limited settings micro-volume methods for clinical chemistry/haematology are not available. Second, before blood sampling by venipuncture, Hb can be determined on capillary blood, ideally by a bedside point-of-care method (such as HemoCue^®^, HemoCue AB, Ängelhom, Sweden), or alternatively by a more cost-effective (but laboratory-bound) capillary haematocrit method. One might also consider assessing children for falciparum malaria before sampling. However, although in the present study severe anaemia was associated with a positive RDT, the RDT result by itself did not accurately predict possible violation of the safety guideline. Researchers should also consider alternative methods for sample collection. As an example, in case plasma is needed, it could be considered to collect a dried blood spot, which implies that a smaller amount of blood needs to be collected. Last and very important, research teams should be aware of the high proportions of severe anaemia among children in sub-Saharan Africa and training and supervision of clinical research staff should emphasize recognition of signs of severe anaemia and indications for blood transfusion. Children suspected of severe anaemia should either be excluded from additional sampling or, in view of the life-saving potential of some diagnostics, be sampled only when blood is available for transfusion. As the proposed guideline is based on limited evidence, future studies should attempt to assess the impact of blood sampling for research in children, especially in *P. falciparum* endemic areas.

## Conclusion

The present analysis shows that the blood volume safety guideline of 3.8 % as proposed by Howie may easily be violated when considering the functional total blood volume in a cohort of febrile children in Burkina Faso. This was related to a high prevalence of (severe) anaemia in the study cohort, associated with current or recent falciparum malaria. When considering the sampling of additional blood for research in children in sub-Saharan Africa, researchers should carefully assess the volume of blood to be sampled and study protocols should include appropriate precautions with regard to patient safety.

## Abbreviations

CRUN: Clinical Research Unit Of Nanoro
Hb: haemoglobin
HRP2: histidine-rich protein-2
NICD: National Institute of Communicable Diseases
RDT: rapid diagnostic test
TBV: total blood volume
TBVf: functional total blood volume
TBVe: estimated total blood volume
UK NEQAS: UK National External Quality Assessment Service
